# Identity-Based Key Exchange on In-Vehicle Networks: CAN-FD & FlexRay

**DOI:** 10.3390/s19224919

**Published:** 2019-11-12

**Authors:** Bogdan Groza, Pal-Stefan Murvay

**Affiliations:** Faculty of Automatics and Computers, Politehnica University of Timisoara, Timisoara 300223, Romania; pal-stefan.murvay@aut.upt.ro

**Keywords:** authentication, CAN bus, identity-based cryptography, key-exchange protocols

## Abstract

Security has become critical for in-vehicle networks as they carry safety-critical data from various components, e.g., sensors or actuators, and current research proposals were quick to react with cryptographic protocols designed for in-vehicle buses, e.g., CAN (Controller Area Network). Obviously, the majority of existing proposals are built on cryptographic primitives that rely on a secret shared key. However, how to share such a secret key is less obvious due to numerous practical constraints. In this work, we explore in a comparative manner several approaches based on a group extension of the Diffie–Hellman key-exchange protocol and identity-based authenticated key agreements. We discuss approaches based on conventional signatures and identity-based signatures, garnering advantages from bilinear pairings that open road to several well-known cryptographic constructions: short signatures, the tripartite Diffie–Hellman key exchange and identity-based signatures or key exchanges. Pairing-based cryptographic primitives do not come computationally cheap, but they offer more flexibility that leads to constructive advantages. To further improve on performance, we also account for pairing-free identity-based key exchange protocols that do not require expensive pairing operations nor explicit signing of the key material. We present both computational results on automotive-grade controllers as well as bandwidth simulations with industry-standard tools, i.e., CANoe, on modern in-vehicle buses CAN-FD and FlexRay.

## 1. Introduction and Motivation

By default, the CAN bus, its newer embodiment with flexible data-rates CAN-FD and the high-end FlexRay bus have no intrinsic security mechanisms. The research community did quickly react to proven threats with countless proposals for assuring security on the CAN bus [1]. The industry has also responded by including specifications for security in the AUTOSAR standards [2]. But while the majority of existing proposals are based on cryptographic Message Authentication Codes (MACs) that rely on a shared secret key, how to share this secret key is an issue that remains largely unaddressed. Notable exceptions are the works in [3,4] which use physical properties of the bus to secretly exchange cryptographic keys. But using physical properties of the bus may further lead to other vulnerabities, e.g., probing as shown in [5], and these approaches do not build on traditional, well-known and understood, cryptographic building blocks. The majority of security protocols from practice rely on a secret key that is exchanged over an insecure network using some public-key mechanism. Automotive networks will be no exception in adhering to this (one alternative is to hard code the keys inside each component which is both insecure and impractical). Clearly, it is much more convenient to be able to link each ECU to a particular manufacturer and to authorize it as part of a network by using the public key of the OEM. However, the PKI (Public-Key Infrastructure) is not universally available inside cars and moreover, exchanging public-key certificates on in-vehicle buses may give rise to additional concerns due to bandwidth limitations. In this work we discuss several approaches in a comparative manner, including identity-based schemes which remove the need for public-key certificates.

**Figure 1:**
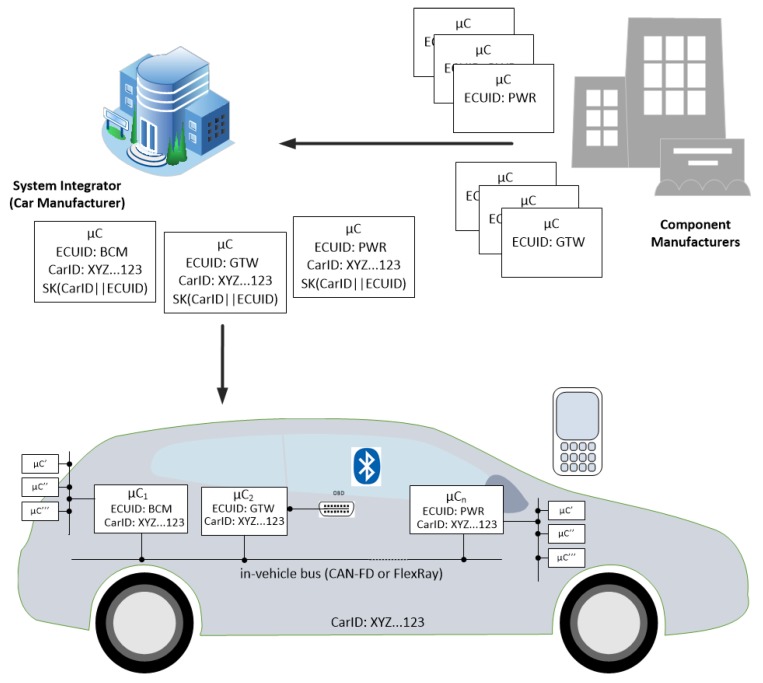
Addressed scenario: car setup during manufacturing.

**Figure 2:**
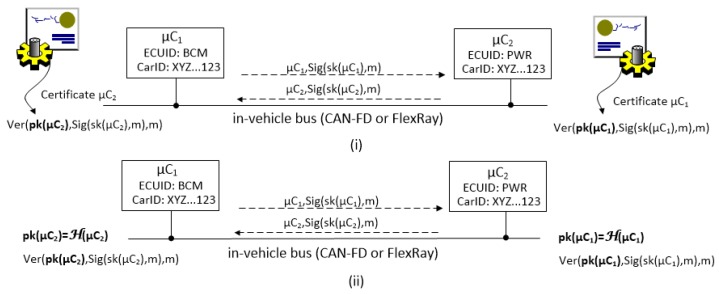
Exchanging signed messages in the conventional (PKI-based) setup (**i**) and in the identity-based setup (**ii**).

The scenario that we address is suggested in Figure 1. Components, i.e., Electronic Control Units (ECUs) originating from the manufacturer arrive at the system integrator and are placed inside the car. To avoid issues that arise from the use of the PKI, each car has a unique identifier (which is consistent to real-world scenarios) and the ECUs have specific names according to the functionality that they address, e.g., BCM (Body Control Module), Gateway (GTW) or Powertrain (PWR). The identifier is enough for deriving the public key in an identity-based scenario and thus it is sufficient for exchanging session keys, secure tunneling, etc. This is suggested in Figure 2 where two nodes exchange signed messages and require the certificates on each side in the conventional setting (i) while the certificates are eliminated in the identity-based setup (ii) where the public key can be derived directly from the identity of the principal (here we assume that derivation is done by some one-way function *H* applied over the identity of the specific microcontroller μC). Some identity-based cryptographic schemes come at higher computational costs, but we show that high-end ECUs can handle them. We assume that these ECUs are linked by CAN-FD and/or FlexRay buses which are capable of handling larger data frames (larger than the traditional 64 bits of CAN). Many other ECUs inside the car can be linked over sub-networks separating them by the vehicle subsystems: body, powertrain, chassis, etc. Mobile devices may be present as they may be linked in the future to diagnostic applications or for remote software updates, etc. Due to a clear trend in bringing Android inside vehicles, e.g., infotainment units, we do not exclude the presence of smart mobile devices as actors in such networks.

Our work discusses several protocol versions since each of these offers certain advantages that are garnered from the underlying cryptographic constructions, e.g., short signatures, the tripartite Diffie–Hellman key-exchange, identity-based signatures and finally identity-based key exchanges. To the best of our knowledge, there is no work to evaluate such key-exchange protocols for in-vehicle communication scenarios. So rather than focusing on designing new cryptographic constructions, our work is concerned with depicting applications of existing protocols and evaluating their performance for in-vehicle environments. Such an analysis is necessary as it offers a first view over the advantages and feasibility of these cryptographic constructions that have not been previously considered in this setting. As already stated, key exchange is an important functionality since all protocols for assuring the security of in-vehicle networks finally depend on a secret shared key.

All of the constructions that we use are based on the Diffie–Hellman key exchange which is the de-facto standard in computer security along with RSA, but each protocol version is distinct in its own way as we discuss later. RSA keys are too large for our embedded systems scenario, e.g., 2048–4096 bits, thus we exclude RSA from our constructions. The protocol versions that we discuss are the following: a regular Diffie–Hellman-based key exchange with short BLS signatures [6], then we replace BLS with an identity-based signature from Paterson [7] and also include the tripartite version [8] of the Diffie–Hellman key exchange. To remove the need for digital signatures, we further use Wang’s key exchange [9] which is also identity-based but perhaps less popular than the rest (it proves however quite efficient and well suited for our scenario). We go even further and to remove the need for the more expensive pairing operations, we also test the identity-based key exchange by Cao et al. [10]. The implementation of these protocols on our experimental automotive-grade controllers is made feasible by the state-of-the-art cryptographic library MIRACL [11].

The design goals of the protocols that we discuss are common characteristics of most Diffie–Hellman-based key-exchange protocols such as authenticity for the secret shared key (which also implies freshness) or forward secrecy (which implies that past sessions are protected even if some future secret key is leaked). We do of course target minimal computational delays and minimal bandwidth usage, but we underline from the beginning that public-key cryptography and identity-based cryptography do not come cheap. An additional flavor that can be added to key-exchange protocols on in-vehicle networks is that session keys can be destroyed in real time on the bus in case something goes wrong with the key-exchange. The idea of destroying unauthentic CAN messages by error flags was first proposed by the work in [12] and it seems a natural design choice in case wrong key-parts are injected (likely due to an adversarial intervention).

### 1.1. Brief Background on CAN-FD & FlexRay

Regular CAN frames may carry at most 64 bits of data, making the traditional CAN not very useful for the protocols that we discuss in this work. The newer CAN-FD or FlexRay have frames that can carry up to 64 and 254 bytes, respectively. These buses are the main target of our work. The approaches that we present can be ported on CAN, but this is not a main intention for our work.

A few words on the structure of CAN and FlexRay frames follow. The first and last fields in each CAN/CAN-FD frame are dedicated to signal the start and end of the frame. In the case of CAN and CAN-FD the SOF (start of frame) indicates the frame start while the EOF (end of frame) indicates the frame end. Similarly, FlexRay uses FSS (frame start sequence) to denote the frame start and FES (frame end sequence) for the frame end. The frame header, which comprises the fields preceding the data or payload field, holds the frame identifier (ID). CAN and CAN-FD both have an ID which has 11 bits in standard frames and 29 bits in extended frames while the FlexRay ID field is always 11 bits long. Moreover, the ID each frame contains additional fields specifying the payload length and type of the frame. FlexRay contains two additional fields dedicated to the header cyclic redundancy check (CRC) and communication cycle count. As previously mentioned, the data/payload field is at most 8 bytes for CAN, 64 bytes for CAN-FD and 254 bytes in the case of FlexRay. All frames also carry a CRC field computed over the entire frame. CAN and CAN-FD contain an additional acknowledge bit used to signal the transmitter that the frame was received by other nodes.

### 1.2. Relevance of Sender/Receiver Identity

In this work we recognize that node identity may prove fundamental in establishing secure connections for certificate-less scenarios, i.e., scenarios where the PKI is absent, which may be common for in-vehicle networks. The brief discussion in this subsection tries to emphasize that node identity is in fact used in many CAN-based scenarios (CAN-FD and FlexRay are simply alternatives when CAN does not cover application needs).

Node identity is not directly defined in the specification of protocols like CAN or FlexRay which usually covers only the lower protocol layers (i.e., the physical and data-link layer). The task of defining node identities is deferred to higher layer protocols and it is in fact done by the SAE J1939 [13] commercial vehicle bus protocol, the ISO-TP standard used for diagnosis, or by the DeviceNet protocol used in automation. All these protocols are CAN-based and they provide various schemes for entity identification on CAN using different abstraction levels (e.g., network node or a specific module within a node). While one may argue that these are based on CAN rather than CAN-FD, we consider that it is natural to expect they will soon migrate to CAN-FD due to the same bandwidth limitations that made CAN unsuitable for modern vehicles. Migration to CAN-FD seems only a matter of time and in any case here we only argue that the identity of nodes is a relevant component at the application layer. We briefly show how the identity is formed in each of these CAN-based protocol suites.

In *SAE J1939* each node has a unique address which is always transmitted by a sender as part of the frame ID. This was used in previous work to provide node identity for message authentication as the absence of authentication mechanism can lead to several attacks on J1939 [14]. Similarly, *ISO-TP*, which is used as a base for diagnostics protocols like UDS and OBD-2, uses the CAN ID field to transmit either the sender address or a unique identifier. The unique identifiers are defined by association with a tuple of communication parameters that include the source and target address, for simplicity, we denote these as $\mathrm{ID}({\mathrm{Addr}}_{A},{\mathrm{Addr}}_{B})$. Please note that $\mathrm{ID}\left({\mathrm{Addr}}_{A},{\mathrm{Addr}}_{B}\right)\neq \mathrm{ID}\left({\mathrm{Addr}}_{B},{\mathrm{Addr}}_{A}\right)$. *DeviceNet* nodes are identified by a MAC ID which is also provided by a sender in all transmitted messages either as part of the ID or data field.

**Figure 3:**
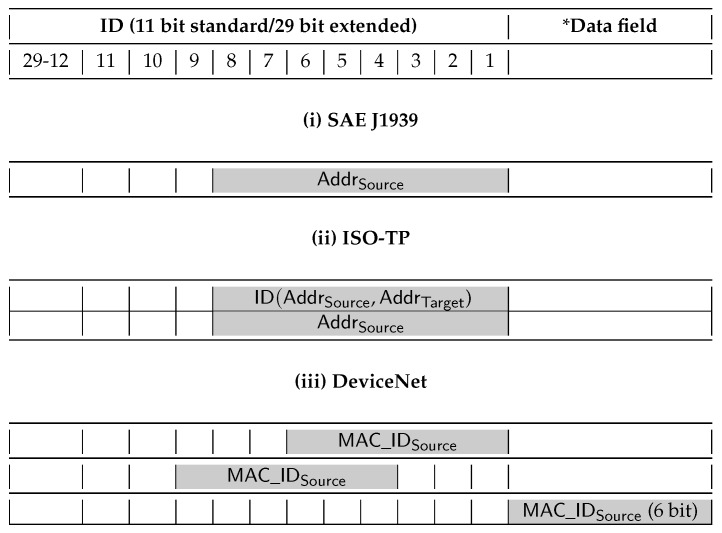
Frame format with node identity as defined by the higher layer protocols of SAE J1939, ISO-TP and DeviceNet.

Figure 3 summarizes the various forms in which senders using one of the previously discussed CAN higher layer protocols encode their identity. Generally, except for a particular case in DeviceNet, node identity is part of the identifier field. This identity, along with specific identifiers for the car and manufacturer may serve as a mean to derive public keys for the components, thus removing the need for PKI.

### 1.3. Related Work on In-Vehicle Network Security

The security of the CAN bus has been addressed by a massive amount of work in the recent years. The research on in-vehicle security was largely motivated by the attacks demonstrated in works such as [15,16], etc. But both the research community and the industry were aware that cars are lacking security for many years before, e.g., one of the earliest works discussing attacks over the CAN bus is [17].

Most of the proposals that can be found in the literature use regular message authentication codes (MACs), e.g., [18,19,20,21], possibly along with time-triggered authentication [22]. Besides regular MACs, hardware countermeasures are also specified in [12] to destroy attacker messages with error flags. Relevant to note, some works focus on efficient signal allocation to fit cryptographic tags along with regular signals inside CAN frames [23,24]. Other recent efforts have also focused on assuring AUTOSAR compliance, e.g., [25], or analyzing trade-offs between safety and security [26].

However, little or no work so far has been focused on how to share keys between CAN nodes. A notable exception is the work in [3,4] which use physical properties of the bus to secretly exchange cryptographic keys. However, as physical properties are harder to control, predict and may succumb to some attacks, e.g., probing [5], relying on secure cryptographic building blocks is a safer alternative. This is the approach we take in our work.

The Diffie–Hellman key exchange [27] is a well understood cryptographic protocol which stays at the foundations of computer security, e.g., it is part of numerous protocols such as SSL/TLS, IPSec, etc. Deriving group keys from the Diffie–Hellman key exchange was probably proposed for the first time in [28]. More recent works such as [29] extend such schemes with dynamic group membership making it possible to add or remove members, partition or merge existing groups. In our scenario we consider that the group is fixed since this is the most likely case om automotive scenarios and we focus on obtaining minimal bus and computational overheads. Designing the schemes for dynamic group membership is a possibility but communication and computational constraints seem a more stringent demand for in-vehicle networks. We also rely on the pairwise key-sharing approach from [28] that propagates over group members in a binary fashion (two by two).

## 2. Key-Exchange Protocols

In what follows we present the protocol versions that we evaluate. We begin with a regular Diffie–Hellman [27]-based key exchange with short BLS signatures [6], then we switch to identity-based signatures due to Paterson [7] and then to Wang’s identity-based key exchange protocol [9]. We also try to reduce the overhead of the Diffie–Hellman protocol by using its tripartite version due to Joux [8]. To remove the need for the more expensive pairing operation we also test the pairing-free identity-based key exchange proposed by Cao in [10]. A brief comparison between the protocols, summarizing their pros and cons, is presented in Table 1 but more details follow upon each protocol description.

### 2.1. Cryptographic Tool-Set

A bilinear pairing is a function $e:G_{1}\times G_{2}\to G_{T}$, where $G_{1},G_{2},G_{T}$ are three cyclic groups, the latter being called the target group. If $G_{1}=G_{2}$ the pairing is called symmetric. The function has three properties which in case when $G_{1}\times G_{2}$ are additive groups can be described as follows: (i) it is bilinear by which $e\left(aP,bQ\right)=e{\left(P,Q\right)}^{ab},\forall P\in G_{1},Q\in G_{2},a,b\in Z$, (ii) non-degenerate which means $\exists P\in G_{1},Q\in G_{2}$ such that e(P,Q)≠1 and (iii) $\forall P\in G_{1},Q\in G_{2}$ the function e(P,Q) is efficiently computable. By bilinearity it immediately follows that $e\left(nP,Q\right)=e\left(P,nQ\right)=e{\left(P,Q\right)}^{n}$. This property stays at the core of several unique cryptographic constructions, out of which we use the following: the tripartite Diffie–Hellman key exchange due to Joux [8], the Boneh–Lynn–Shacham short signature scheme [6], the identity-based signature from Paterson [7], the identity-based key exchange of Wang [9]. For clarity, we briefly recap these building blocks, for additional details we refer the reader to the original works describing these protocols.

*Boneh–Lynn–Shacham short signature scheme*. The short signature in [6] sets room for signatures that are merely 160 bits in length. The scheme is one of the most immediate and intuitive application of pairings. By setting the public key to (αP,P), where α is a random secret, the signature is computed as $s=H{\left(m\right)}^{\alpha }$. To verify that the signature is correct, the verifier simply checks that e(αP,H(m))=e(P,s).

*Identity-based signature by Paterson.* In [7] an identity-based signature is immediately derived from pairings. A public key is set for each user as $Q_{I\;D}=H\left(I\;D\right)$ by computing hash function *H* over the identity ID, the private key is $d_{I\;D}=\alpha Q_{I\;D}$ and α is the secret master key. Values P,αP are also made public as system parameters. The signature is the pair R,S where $R=kP,S=k^{-1}\left(H_{2}\left(m\right)P+H_{3}\left(R\right)d_{I\;D}\right)$. Verification implies checking for the following equality: $e\left(R,S\right)=e{\left(P,P\right)}^{H_{2}\left(m\right)}e{\left(\alpha P,Q_{I\;D}\right)}^{H_{3}\left(R\right)}$. Indeed, by looking at the second term from the right $e{\left(\alpha P,Q_{I\;D}\right)}^{H_{3}\left(R\right)}=e{\left(P,\alpha Q_{I\;D}\right)}^{H_{3}\left(R\right)}=e{\left(P,d_{I\;D}\right)}^{H_{3}\left(R\right)}=e\left(P,H_{3}\left(R\right)d_{I\;D}\right)$. Thus, the second term is $e{\left(P,P\right)}^{H_{2}\left(m\right)}e\left(P,H_{3}\left(R\right)d_{I\;D}\right)=e\left(P,H_{2}\left(m\right)P+H_{3}\left(R\right)d_{I\;D}\right)$ and the left term is the same since $e\left(R,S\right)=e\left(kP,k^{-1}\left(H_{2}\left(m\right)P+H_{3}\left(R\right)d_{I\;D}\right)\right)=e\left(P,H_{2}\left(m\right)P+H_{3}\left(R\right)d_{I\;D}\right)$.

*Tripartite Diffie–Hellman.* Joux [8] generalizes the Diffie–Hellman key exchange for the case of the three parties. It can be immediately observed that from the Diffie–Hellman key shares of three parties aP,bP,cP, a unique shared key can be computed as $e{\left(bP,cP\right)}^{a}=e{\left(aP,cP\right)}^{b}=e{\left(aP,bP\right)}^{c}=e{\left(P,P\right)}^{abc}$. Without bilinear pairings, in order to compute the common shared key abcP, the parties will also need to exchange abP,cbP,bcP, besides the regular aP,bP,cP. The tripartite Diffie–Hellman key exchanges makes this feasible without the expense of the three additional messages.

*Wang identity-based authenticated key exchange.* Wang [9] proposes an efficient identity-based key exchange protocol. The following values are set: point $G_{I\;D}=H\left(I\;D\right)$ is extracted by computing hash function *H* over the identity ID of some principal, the private key of each party is $d_{I\;D}=\alpha G_{I\;D}$ and α is the secret master key. Now, from the Diffie–Hellman key shares $aG_{I\;D_{A}}$ and $bG_{I\;D_{B}}$ (generated by principal A and B which randomly select *a* and *b*) a common session key can be established as: $e{\left(G_{I\;D_{A}},G_{I\;D_{B}}\right)}^{\left(a+s_{A}\right)\left(b+s_{B}\right)}=$$e(\left(a+s_{A}\right)d_{I\;D_{A}},s_{B}G_{I\;D_{B}}+bG_{I\;D_{B}})=$$e(s_{A}G_{I\;D_{A}}+aG_{I\;D_{A}},$$\left(b+s_{B}\right)d_{I\;D_{B}})$. where $s_{A}=\pi \left(aG_{I\;D_{A}},bG_{I\;D_{B}}\right)$ and $s_{B}=\pi \left(aG_{I\;D_{B}},bG_{I\;D_{A}}\right)$ for some hash function π.

*Cao et al. identity-based key exchange.* The work in [10] proposes an identity-based key exchange that does not rely on the expensive pairing operation e(·,·). The protocol assumes a master secret key $x\in Z_{p}^{*}$ and a master public key xP where *P* is a point of an elliptical-curve $E/F_{p}$. To compute the secret key of a user $I\;D_{A}$, the key-generation center selects random $r\in Z_{p}$, computes $R_{I\;D_{A}}=rP$, $h=H(I\;D_{A},R_{I\;D_{A}})$ and $s_{I\;D_{A}}=r+hx$ (where *H* is a hash functions). The pair $\left(s_{I\;D_{A}},R_{I\;D_{A}}\right)$ is the user secret key. The same is done for the other user $I\;D_{B}$. To exchange a key, users $I\;D_{A}$ and $I\;D_{B}$, generate a random secret value *a* and *b* respectively, the compute $T_{I\;D_{A}}=aP$ and $T_{I\;D_{A}}=bp$. Subsequently, they exchange $R_{I\;D_{A}},T_{I\;D_{A}}$. The common secret key is derived from the two identical shares $K_{I\;D_{A}I\;D_{B}}^{1}=s_{I\;D_{A}}T_{I\;D_{A}}+a\left(R_{I\;D_{B}}+H\left(I\;D_{B},R_{I\;D_{B}}\right)xP\right)=s_{I\;D_{B}}T_{I\;D_{B}}+b\left(R_{I\;D_{A}}+H\left(I\;D_{A},R_{I\;D_{A}}\right)xP\right)=K_{I\;D_{B}I\;D_{A}}^{1}$ and $K_{I\;D_{A}I\;D_{B}}^{2}=aT_{I\;D_{B}}=bT_{I\;D_{A}}=K_{I\;D_{B}I\;D_{A}}^{2}$ as $sk=H\left(I\;D_{A},I\;D_{B},T_{I\;D_{A}},T_{I\;D_{B}},K_{I\;D_{A}I\;D_{B}}^{1},K_{I\;D_{A}I\;D_{B}}^{2}\right)=H\left(I\;D_{A},I\;D_{B},T_{I\;D_{A}},T_{I\;D_{B}},K_{I\;D_{B}I\;D_{A}}^{1},K_{I\;D_{B}I\;D_{A}}^{2}\right)$.

### 2.2. PKI-Based Protocol Version, with BLS Signatures

In case of the BLS signature scheme [6] the public key cannot be retrieved from the identity of the principals (this is not an identity-based scheme). Thus, the following scheme requires certificates and a mean to distribute them. Certificates will not be sent during each key exchange, since they may be valid for longer periods, and revocation lists or certificate updates may be performed periodically or when requested by the manufacturer. We include this scheme mainly as a baseline for comparison to traditional PKI-based approaches. Another relevant reason for exploring it is the fact that it offers the shortest signatures that are known, i.e., 160 bits. We present all protocol versions following a syntax of Send and Extract events. The first event consists of sending the key-part that is shared by each ECU and the second consists of the extraction of the common session key that was so far negotiated, i.e., after each ECU sent his share. Additionally, a Stop event can be invoked to stop the protocol run in case that any ECU notices inconsistencies in the key that was so far negotiated.

*Protocol 1 (Diffie–Hellman with short BLS signatures—DH-BLS).* We assume the existence of a publicly known key derivation function KD. In what follows $\mathrm{Sig}({\mathrm{ECU}}_{i},m)$ denotes a signature performed with the private key of principal ${\mathrm{ECU}}_{i},i=1\ldots n$ on message *m*, i.e., the first term of the Sig function denotes the owner of the private key. Each ${\mathrm{ECU}}_{i},i=1\ldots n$ runs the following three procedures:

1. $\mathrm{SendDH}({\mathrm{ECU}}_{i})$ in which ${\mathrm{ECU}}_{i=1\ldots n}$ sends its Diffie–Hellman key share as follows: generates a random value $a_{i}$ and computes $a_{i}P$ then computes the coefficient $a_{1⊳i}=\mathrm{KD}\left(a_{i}a_{1⊳i-1}P\right)$, where $a_{1⊳i-1}P$ is the value previously broadcast by ${\mathrm{ECU}}_{i-1}$ (${\mathrm{ECU}}_{1}$ and ${\mathrm{ECU}}_{n}$ will skip the computation of $a_{1⊳i}P$), and broadcasts the tuple $\left\{a_{i}P,a_{1⊳i}P,\mathrm{Sig}({\mathrm{ECU}}_{i},a_{i}P||a_{1⊳i}P)\right\}$ on the bus (here the signature is instantiated by the BLS scheme [6]),
2. ExtractDH in which ${\mathrm{ECU}}_{i=1\ldots n}$ retrieves the common secret key as follows: using subsequent values broadcast on the bus by ${\mathrm{ECU}}_{j=i+1\ldots n}$, at each newly received key share $\left\{a_{j}P,a_{1⊳j}P\right\}$ it computes the new coefficient $a_{1⊳j}=\mathrm{KD}\left(a_{1⊳j-1}a_{j}P\right)$,
3. StopDH at each protocol step, any of the ${\mathrm{ECU}}_{i=1\ldots n}$ when computing $a_{1⊳j},j>i$, if the newly computed value multiplied by point *P* does not match the received value $a_{1⊳j}P$ the protocol is aborted and this is signaled by error flags.

**Figure 4:**
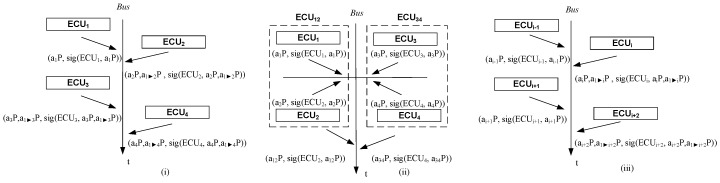
Overview of protocol procedures for DH-BLS (**i**), pairwise version of DH-BLS (**ii**) and tripartite Diffie–Hellman (**iii**).

The common session key is $a_{1⊳n}P=\mathrm{KD}\left(a_{1⊳n-1}a_{n}P\right)$. The protocol stops only when signatures on the session key have been correctly retrieved from each node. Otherwise, as well as in case of errors, the protocol restarts from step 1. The protocol run between the first 4 nodes is suggested in Figure 4 part (i). Please note that ${\mathrm{ECU}}_{1}$ does not compute $a_{1⊳1}P$ since there are no previous Diffie–Hellman shares and similarly ${\mathrm{ECU}}_{n}$ will not compute $a_{1⊳n}P$ since there is not further node to exchange keys with.

To reduce delays due to intensive computations, we may add some parallelism by grouping nodes two by two and the exchange the key between the logical entities formed by each two nodes. This pairwise approach is suggested for the case of 4 nodes in Figure 4 part (ii). The exchange between ${\mathrm{ECU}}_{1}$, ${\mathrm{ECU}}_{2}$ and ${\mathrm{ECU}}_{3}$, ${\mathrm{ECU}}_{4}$ are encircled since the computations of ${\mathrm{ECU}}_{1}$ and ${\mathrm{ECU}}_{3}$ are done in parallel (note that in part (i) of the figure the computations propagate sequentially). Subsequently, the logical entities ${\mathrm{ECU}}_{12}$ and ${\mathrm{ECU}}_{34}$ may continue to exchange a new session key. We consider that this is done either by ${\mathrm{ECU}}_{1}$ or ${\mathrm{ECU}}_{2}$ on behalf of ${\mathrm{ECU}}_{12}$ or by ${\mathrm{ECU}}_{3}$ or ${\mathrm{ECU}}_{4}$ on behalf of ${\mathrm{ECU}}_{34}$. For brevity we skip the formalism for this protocol version. This variation is also possible for two of the schemes that follow.

### 2.3. Identity-Based Protocol Versions

The following protocol versions rely on the identities of principals which can be immediately derived from the unique name of the ECU and the identifier selected by the manufacturer. The identity-based versions of the key exchange do not benefit from the short BLS signatures, but they eliminate the need for public-key certificates. The protocol based on the Paterson [7] signature is identical to the previous BLS [6]-based protocol, it is only the signature that is different. The modification is outlined next.

*Protocol 2 (Diffie–Hellman with Paterson identity-based signature—DH-Pat).* Each ${\mathrm{ECU}}_{i},i=1\ldots n$ runs the procedures of Protocol 1 with the following modification: in step $\mathrm{SendDH}({\mathrm{ECU}}_{i})$ the signature is computed as a Paterson identity-based signature $\mathrm{Sig}({\mathrm{ECU}}_{i},a_{i}P)$ which is the pair {R,S} as outlined in the previous section.

We can optimize this by using the tripartite Diffie–Hellman scheme due to Joux [8] which allows the dropping of one Diffie–Hellman computation for even-numbered nodes. This is described next

*Protocol 3 (Tripartite Diffie–Hellman with Paterson identity-based signature—3DH-Pat).* Each ${\mathrm{ECU}}_{i},i=1\ldots n$ runs the procedures of Protocol 2 with the following modification:

1. in $\mathrm{SendDH}({\mathrm{ECU}}_{i})$ even-numbered ECUs, i.e., ${\mathrm{ECU}}_{i=1\ldots n},i\;\equiv \;0\;\;\;\mathrm{mod}\;2$ sends only their Diffie–Hellman key share, i.e., $a_{i}P$, without the common share $a_{1⊳i}P$, and odd-numbered ECUs, i.e., ${\mathrm{ECU}}_{i=1\ldots n},i\;\equiv \;1\;\;\;\mathrm{mod}\;2$ compute the shared session key with the tripartite Diffie–Hellman (see below) and send the share $a_{1⊳i}P$ (the signature is computed as a Paterson identity-based signature $\mathrm{Sig}({\mathrm{ECU}}_{i},a_{i}P)$, i.e., the pair {R,S}),
2. ExtractDH in which each ${\mathrm{ECU}}_{i=1\ldots n}$ retrieves the common secret key as a regular tripartite Diffie–Hellman key, i.e., even-numbered ECUs compute $e{\left(a_{1⊳i-1}P,a_{i+1}P\right)}^{a_{i}}$, and odd-numbered ECUs compute $e{\left(a_{1⊳i-1}P,a_{i}P\right)}^{a_{i+1}}$.

Figure 4 part (iii) clarifies the key-extraction starting from the case of an even-index node ${\mathrm{ECU}}_{i-1}$. The even-numbered nodes ${\mathrm{ECU}}_{i-1}$ and ${\mathrm{ECU}}_{i+1}$ do not have to compute a common key. Only odd-numbered nodes ${\mathrm{ECU}}_{i}$ and ${\mathrm{ECU}}_{i+2}$ must compute $a_{1⊳i}P$ and $a_{1⊳i+2}P$ respectively.

Grouping the nodes two by two to achieve some parallelism in computation is possible in the case of the first Paterson-based scheme, i.e., Protocol 2. For its tripartite version, i.e., Protocol 3, the nodes may be grouped three-by-three. For brevity, we skip formalism for these protocol variations, but we do discuss them later in the experimental section and evaluate their performance. Such a variation is not possible for the following scheme.

The following protocol uses Wang identity-based key exchange [9]. The protocol is similar to Protocols 1, 2 and 3 in which the Diffie–Hellman shared key is replaced with Wang’s identity-based share. Note however that the protocol now establishes only a pairwise shared key between each two ECUs. We add a symmetric-key-exchange step later to establish a common key for all nodes.

*Protocol 4 (Wang identity-based).* Each ${\mathrm{ECU}}_{i},i=1\ldots n$ runs the following the procedures:

1. $\mathrm{SendWang}({\mathrm{ECU}}_{i})$ in which ${\mathrm{ECU}}_{i=1\ldots n}$ sends the key share required by the Wang key-exchange protocol, i.e., $a_{i}G_{I\;D({\mathrm{ECU}}_{i})}$,
2. ExtractWang in which each ${\mathrm{ECU}}_{i=1\ldots n}$ retrieves the common secret key with ${\mathrm{ECU}}_{j=1\ldots n},j\neq i$ as $e{\left(G_{I\;D({\mathrm{ECU}}_{i})},G_{I\;D({\mathrm{ECU}}_{j})}\right)}^{\left(a+s\left({\mathrm{ECU}}_{i}\right)\right)\left(b+s\left({\mathrm{ECU}}_{j}\right)\right)}$.

We describe next the version based on Cao et al. [10] identity-based key exchange. Similar to the protocol version based on Wang [9], the protocol establishes a pairwise shared key between each two ECUs.

*Protocol 5 (Cao et al. identity-based).* Each ${\mathrm{ECU}}_{i},i=1\ldots n$ runs the following the procedures:

1. $\mathrm{SendCao}({\mathrm{ECU}}_{i})$ in which ${\mathrm{ECU}}_{i=1\ldots n}$ sends the key share required by the Cao key-exchange protocol, i.e., $R_{I\;D({\mathrm{ECU}}_{i})}$ and $T_{I\;D({\mathrm{ECU}}_{i})}=a_{i}P$,
2. ExtractCao in which each ${\mathrm{ECU}}_{i=1\ldots n}$ retrieves the common secret key with ${\mathrm{ECU}}_{j=1\ldots n},j\neq i$ as $K_{I\;D\left({\mathrm{ECU}}_{i}\right),I\;D\left({\mathrm{ECU}}_{j}\right)}^{1}=s_{I\;D({\mathrm{ECU}}_{i})}T_{I\;D({\mathrm{ECU}}_{i})}+a_{i}\left(R_{I\;D({\mathrm{ECU}}_{j})}+H\left(I\;D\left({\mathrm{ECU}}_{j}\right),R_{I\;D({\mathrm{ECU}}_{j})}\right)xP\right)$.

*Symmetric-key-exchange for a common session key.* The previous two protocols do not allow an immediate extension of the Diffie–Hellman key exchange to groups of nodes, they only lead to a pairwise shared key between each two nodes on the network. However, this can be easily extended, with one additional protocol step based on symmetric-key primitives that facilitates the establishment of a unique session key between all nodes. In order to establish the common session key, each node may broadcast a packet containing his share of the symmetric key, encrypted and authenticated by a MAC, computed for each other node with the common key. That is, let $k_{i,j}$ be the key shared between ${\mathrm{ECU}}_{i}$ and ${\mathrm{ECU}}_{j}$ (generated with either the Wang or Cao-based protocol versions), then the packet sent by ${\mathrm{ECU}}_{i}$ will contain the encryption and MAC of $k_{i}^{\prime }$ with each of the shared keys, i.e., $E_{{\mathrm{KD}}^{\prime }\left(k_{i,1}\right)}\left(k_{i}^{\prime }\right),{\mathrm{MAC}}_{{\mathrm{KD}}^{\prime \prime }\left(k_{i,1}\right)}\left(k_{i}^{\prime }\right)\left|\right|E_{{\mathrm{KD}}^{\prime }\left(k_{i,2}\right)}\left(k_{i}^{\prime }\right),$${\mathrm{MAC}}_{{\mathrm{KD}}^{\prime \prime }\left(k_{i,2}\right)}\left(k_{i}^{\prime }\right)\left|\right|$…$\left|\right|E_{{\mathrm{KD}}^{\prime }\left(k_{i},n\right)}\left(k_{i}^{\prime }\right)$, ${\mathrm{MAC}}_{{\mathrm{KD}}^{\prime \prime }\left(k_{i,n}\right)}\left(k_{i}^{\prime }\right)$. Here $k_{i}^{\prime }$ is the part of the common session key generated by ${\mathrm{ECU}}_{i}$. The common session key is further derived as $\mathrm{KD}(k_{n}^{\prime },\ldots \mathrm{KD}\left(k_{2}^{\prime },\mathrm{KD}\left(k_{1}^{\prime },0\right)\right)\ldots )$. This procedure can be summarized in the following two protocol steps:

1. $\mathrm{SendSym}({\mathrm{ECU}}_{i})$ in which ${\mathrm{ECU}}_{i=1\ldots n}$ proceed in consecutive order by sending message $E_{{\mathrm{KD}}^{\prime }\left(k_{i,1}\right)}\left(k_{i}^{\prime }\right),{\mathrm{MAC}}_{{\mathrm{KD}}^{\prime \prime }\left(k_{i,1}\right)}\left(k_{i}^{\prime }\right)\left|\right|$$E_{{\mathrm{KD}}^{\prime }\left(k_{i,2}\right)}\left(k_{i}^{\prime }\right),$${\mathrm{MAC}}_{{\mathrm{KD}}^{\prime \prime }\left(k_{i,2}\right)}\left(k_{i}^{\prime }\right)\left|\right|$…$\left|\right|E_{{\mathrm{KD}}^{\prime }\left(k_{i},n\right)}\left(k_{i}^{\prime }\right),$${\mathrm{MAC}}_{{\mathrm{KD}}^{\prime \prime }\left(k_{i,n}\right)}\left(k_{i}^{\prime }\right)$ in which $k_{i}^{\prime }$ is symmetric key generated by ${\mathrm{ECU}}_{i}$ (the size of $k_{i}$ is to be determined by practical constraints, more discussions follow),
2. $\mathrm{ExtractSym}({\mathrm{ECU}}_{i})$ in which every ${\mathrm{ECU}}_{i},i=1\ldots n$ retrieves the symmetric key from all other nodes by decrypting the packets from $\mathrm{SendSym}({\mathrm{ECU}}_{i})$ and then computes the common session key as $\mathrm{KD}(k_{n},\ldots \mathrm{KD}\left(k_{2},\mathrm{KD}\left(k_{1},0\right)\right)\ldots )$.

Due to the limited size of the payload, the encryption and MAC may be limited to the number of bits in the data field divided by the number of nodes. For example, in case of 512 bit frames and 8 nodes, each node will send 64 bits of key material for each of the other nodes, i.e., 32 bits for the key and 32 bits for the MAC. While 32 bits is insufficient for a single MAC, there are 8 such MACs in the packet and the adversary must be able to forge more than a single MAC to forge a common session key.

### 2.4. Security Discussion

We do assume the existence of a regular adversary that has full control over the communication channel. As stated, our protocols rely on well-known cryptographic blocks and deriving security proofs would be out of scope since our goal is to establish whether these protocols are suitable for in-vehicle scenarios. In particular, the first three schemes that we discuss are based on signed versions of the Diffie–Hellman key agreement, which have a well understood security and stay at the core of many protocols from practice, e.g., SSL/TLS, being studied in the literature long ago, e.g., [30]. The last two protocols that we evaluate, i.e., Wang [9] and Cao’s IBKE [10], are indeed more recent, but they are both accompanied by security proofs in the original works. If flaws and fixes are found in the future, then these should be easy to embed in the constructions from this work. In what follows, we give only a brief discussion on specific security concerns with respect to our setting.

Due to computational and bandwidth constraints, the key is established in a bandwagon manner, i.e., the second node of each two nodes that already exchanged a Diffie–Hellman key will continue negotiations for a new key with the next node (or pair of nodes in the pairwise version of the scheme). Because of this procedure, if the i-th node is malicious and has an active role, it may inject a session key that does not match the correct key $a_{1⊳i}P$ (note that this node must still be a genuine node of the network since he still needs to sign the message). If such a situation occurs, the passive node can destroy the following session keys with error flags forcing the key exchange to restart. If the attacker is also able to disconnect the node from the bus immediately after it sends his Diffie–Hellman key share, then the best that the adversary could achieve is a DoS since the disconnected node will not know the session key. This can be again fixed by triggering a re-negotiation of the session key. In an automotive scenario however, it is likely that all ECUs are trusted and the case of a malicious ECU that injects wrong session keys appears less likely and will have little benefits for the adversary. Only a DoS will be caused and if the malicious node is also a genuine node from the network it will have the secret shared key anyway since it is included in the key exchange protocol. Consequently, such an attack seems to have quite a limited impact. Nonetheless, DoS attacks on the CAN bus are generally feasible due to the design of the bus, i.e., dominant bits always overwrite recessive ones, and these cannot be stopped by cryptographic countermeasures. Thus, we consider such attacks to be out of scope for the current work.

## 3. Experimental Results

**Figure 5:**
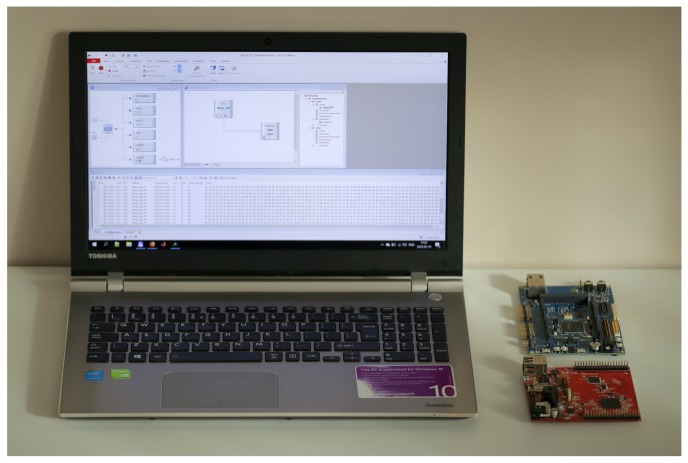
Components employed from our experiments.

This section discusses computational and bandwidth results for each of the protocols. The experiments were performed using high-end embedded platforms for computational results as well as the CANoe simulation environment for bandwidth results, devices are illustrated in Figure 5.

### 3.1. Computational Results with Pairing Libraries

For evaluating the computational overhead of the previously proposed key-exchange protocols we selected two automotive-grade platforms with 32-bit cores designed to offer high performance for demanding applications. The first is a Microchip SAM V71 microcontroller built around an ARM Cortex M7 core which can operate at up to 300 MHz and has access to the 384 KB SRAM and 2 MB of Flash on-chip memories. The second, an Aurix TC297 from Infineon, features 3 CPUs based on Infineon’s TriCore technology each running at up to 300MHz with 728 KB of SRAM and 8 MB of Flash.

All the computational measurements were done by clocking the CPUs of the two microcontrollers at their maximum frequency, i.e., 300 MHz. Only one core was used on the TC297 platform with no task parallelization for a fair comparison of performance measurements on the two platforms. Also, the same optimization options for the GNU compilers were used to generate the object code for the two platforms.

For evaluating the key exchange mechanisms on the two embedded platforms we used MIRACL (Multiprecision Integer and Rational Arithmetic C Library) [11], an open source C library with support for ECC. The implementations of the cryptographic building blocks for the protocols were executed on each of the two employed microcontrollers and the run times of the main operations were measured. The MIRACL [11] cryptographic library provides examples for BLS and Wang. However, it does not have an implementation for Paterson’s scheme, neither for the tripartite Diffie–Hellman or Cao key exchanges, which we had to implement ourselves and was not a difficult task given the extensive support of the library.

The BLS signature was evaluated on a 20 byte curve, i.e., option MR_PAIRING_MNT, which yields a short 20-byte signature when coordinates are in compressed form (an extra bit is needed for the sign). The BLS signature was done on type 3 pairings. Both the Paterson identity-based signature and the Wang identity-based key exchange require type 1 pairings. We evaluated them both on GF2 with option MR_PAIRING_SS2 which lead to 48 byte coordinates, i.e., 392-bit curves, and $Z_{p}$ with option MR_PAIRING_SSP which resulted in 64 byte coordinates, i.e., 512-bit curves. For Paterson’s scheme the size of the signature is double since there are two values in each signature.

Table 2 summarizes these run times measured in milliseconds. The Infineon TC297 generally outperforms the Microchip core. As for the signature schemes, Paterson gave poorer results than BLS. Thus, it seems that BLS or Wang’s key exchange are the preferred solution. Pairings over GF2 are also included for comparison but they are known to be insecure so they will not be present in the synthetic analysis from the next section. The results in Table 2 do not account for specific optimizations in the MIRACL library which may further improve on the computational time.

To obtain better performance results we further implemented the steps required for the Cao-based protocol version. The implementation was done both on the fast and small 160-bit non-pairing friendly curve but also on the 512-bit pairing friendly curve to obtain clear image on computational penalties. Of course, the implementation on the pairing friendly curve does not use the expensive pairing operation, but it is still about 4 times slower. On the 160-bit curve, the results are several times (or up to an order of magnitude) faster than for the rest of the primitives. The results are presented in Table 3.

### 3.2. Synthetic Performance Evaluation

In Table 4 we estimated the computational and bus overhead for each of the protocol versions. We use the following notations: (i) $t_{\mathrm{sBLS}},t_{\mathrm{sPat}}$ is the time to sign a message with the two schemes, i.e., BLS and Paterson, (ii) $t_{\mathrm{vBLS}},t_{\mathrm{vPat}}$ is the time to verify a signature with the two schemes, i.e., BLS and Paterson, (iii) $t_{\mathrm{sDH}}$ is the time to compute the Diffie–Hellman key share of a node, i.e., aP, (iv) $t_{\mathrm{eDH}}$ is the time to extract the common Diffie–Hellman key share of a node, i.e., compute abP from *a* and bP, (v) $t_{\mathrm{etDH}}$ is the time to extract the common key share of the tripartite Diffie–Hellman, i.e., compute abcP from a,bP and cP, (vi) $t_{\mathrm{shWang}}$ and $t_{\mathrm{kWang}}$ is the time to compute the Wang key share and the time to extract the common key between two nodes, (vii) $t_{\mathrm{shCao}}$ and $t_{\mathrm{kCao}}$ is the time to compute the Cao key share and the time to extract the common key between two nodes.

*Computational time.*Table 4 presents the synthetic evaluation for computational time and bus overhead. For the Diffie–Hellman-based schemes, each sender node, i.e., ${\mathrm{ECU}}_{i}$ at step *i*, has to verify the signature on the received packet, extract the Diffie–Hellman session key $a_{i}a_{1⊳i-1}P$, compute a new Diffie–Hellman key share $a_{1⊳i}P$ and sign the packet he sends. The first node does not need to extract a common session key and verify the signature since there is no previous sender while the last node does not need to send a secondary key share since no sender follows. We assume that all nodes have computed their Diffie–Hellman key share $a_{i}P$ in parallel at protocol initialization to save on computational time. For all protocols, we have the same sum of the four computational terms for each sender, e.g., in case of the DH-BLS we have $t_{\mathrm{vBLS}}+t_{\mathrm{eDH}}+t_{\mathrm{sDH}}+t_{\mathrm{sBLS}}$ and it follows similarly for the rest. The exceptions are the Wang and Cao-based schemes were each node must compute his share then extract the next key, e.g., $t_{\mathrm{shWang}}+t_{\mathrm{kWang}}$. The time for the symmetric-key extraction in case of the Wang-based scheme is negligible, i.e., under 1ms and it is neglected to avoid overloading the table. For *n* nodes, we sum over the computations for all nodes (the first node is spared from one computation of the common session key and one signature verification). This leads to $n\left(t_{\mathrm{sDH}}+t_{\mathrm{sBLS}}\right)+\left(n-1\right)\left(t_{\mathrm{eDH}}+t_{\mathrm{vBLS}}\right)$ for the DH-BLS scheme and similarly for the rest. With the schemes from Wang and Cao, in case of *n* nodes, there are n−1 key extractions since each node has to extract a key with each of the other nodes, e.g., $nt_{\mathrm{shWang}}+\left(n-1\right)t_{\mathrm{kWang}}$. The pairwise versions further reduce on the computational load for *n* nodes since extraction goes pairwise in parallel (two by two or three by three in case of the tripartite versions), i.e., in case of a single node before each key-exchange computations are identical to the previous case and this goes for $\log_{2}n$ and $\log_{3}n$ steps in case of the tripartite versions. This leads to $\left(t_{\mathrm{sDH}}+t_{\mathrm{eDH}}+t_{\mathrm{sBLS}}+t_{\mathrm{vBLS}}\right)\log_{2}n$ in case of the Pairwise DHKE-BLS and similarly for the rest of the pairwise versions.

*Bus overhead.* Let *ℓ* denote the size of the point on the elliptic curve in bytes (we discuss later practical instantiations for each of the curves). The BLS signature requires only one point for encoding. In case of DH-BLS, the size of the message for the first and last node, i.e., ${\mathrm{ECU}}_{1}$ and ${\mathrm{ECU}}_{n}$, is smaller since it does not include the second Diffie–Hellman key share. This leads to a total of 2ℓ bytes for the first and last nodes. For the rest n−2 nodes, an additional Diffie–Hellman key share is needed which, along with one point of the first Diffie–Hellman keyshare and one for the signature, requires a total of 3ℓ bytes. Summing up for all the *n* nodes, this leads to 4ℓ+3(n−2)ℓ=ℓ(3n+2). In the pairwise version of the DH-BLS scheme just one Diffie–Hellman key share and one signature need to be exchanged by each node, resulting in 2ℓ bytes. Over the entire binary three there are $\left(2^{\log_{2}n+1}-1\right)$ nodes and thus it leads to $2\ell (2^{\log_{2}n+1}-1)$ bytes. Similar computations are done for the DH-Pat protocol version, but here the signature is larger requiring two points from the curve for encoding. For one Diffie–Hellman key share and one signature we have a total of 3ℓ bytes. These are sent by the first and last node. The rest of the n−2 nodes have to send and additional Diffie–Hellman key share leading to 4ℓ and for all of the *n* nodes to a total of 6ℓ+4(n−2)ℓ=2ℓ(2n−1). Similarly for the pairwise version of the DH-Pat scheme we have the larger signature compared to DH-BLSwhich leads to $3\ell (2^{\log_{2}n+1}-1)$ bytes. The tripartite 3DH-Pat version saves one Diffie–Hellman key share for each even node, resulting in a total of $3\ell \frac{n}{2}+4\ell \frac{n}{2}=7\ell \frac{n}{2}$. In the pairwise tripartite version there are $\frac{3^{\log_{3}n+1}-1}{2}$ nodes in the three and since each sends 3ℓ bytes (one key share and one signature) this results in a total of $3\ell (\frac{3^{\log_{3}n+1}-1}{2})$ bytes. Finally, for Wang’s scheme one point of the curve is sent by each node, leading to a total of ℓn bytes. Cao’s scheme requires two points from the curve which leads to a total of 2ℓn bytes.

*Practical instantiations of the curves in MIRACL.* We now discuss the resulting busload after specific curve instantiations available in MIRACL. We assume point compression is used, thus a single coordinate (X) plus the sign of the other (Y) is sufficient. In case of DH-BLS, the size of one coordinate of the point on the elliptic curve is 20 bytes and an extra byte is used for the sign resulting in a total of 21 bytes. The size of the message for the first and last node will be 42 bytes and for the following n−2 nodes 63 bytes. In the pairwise version of the DH-BLS scheme there will be a total of $42(2^{\log_{2}n+1}-1)$ bytes. For the DH-Pat protocol version, which uses a symmetric pairing, we used a 64 byte curve (which was the smallest symmetric pairing we were able to find in the MIRACL library, one additional byte is for the sign of the point). For one Diffie–Hellman key share and one signature we have a total of 3×65=195 bytes. The rest of the n−2 node must send and additional 65 bytes, thus 195+65=260 bytes in total. Similarly for the pairwise version of the DH-Pat scheme we have $195(2^{\log_{2}n+1}-1)$ bytes and for the tripartite 3DH-Pat version $\frac{n}{2}195+\frac{n}{2}260$. For the pairwise tripartite version this leads to a total of $195(\frac{3^{\log_{3}n+1}-1}{2})$ bytes. Finally, for Wang’s scheme we use the same 64 byte curve available in MIRACL leading to a total of 65n bytes.

**Figure 6:**
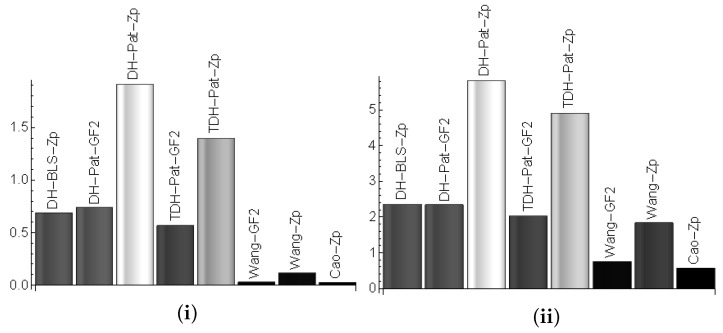
Computational time (s) on one node (**i**) and estimated computational time for 4 nodes (**ii**) (based on Infineon TC297 results).

Figure 6 shows the estimated computational time for one node (left) and for 4 nodes when running each of the protocol versions. The bus time is not added since it is too low compared to the computational time, i.e., the computational cost for each operation is in the order of hundreds of milliseconds while the time to send a packet on the bus in the order of hundreds micro-seconds. For one node the computational time is 1s or less at each step, with the exception of the DH-Pat scheme, and for 4 nodes this increases to around 2 s for all schemes except DH-Patwhich may be too computationally intensive. Such timings are affordable in case key negotiation is done during car setup at a reseller or in an authorized garage during periodic maintenance services. By using the pairing-free version based on Cao’s protocol, the computational time for sharing a key between 4 nodes will drop to around 500ms which is the time required for generating one share and then reconstructing three keys from the shares of the other three nodes.

**Figure 7:**
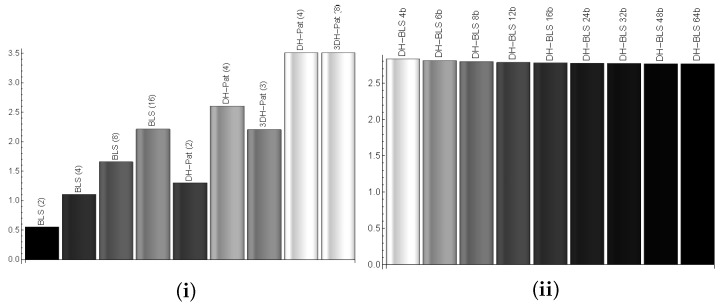
Computational and bus transfer time (s) for: the three versions of the protocol in case of 2, 4, 8 and 16 nodes on CAN-FD (**i**) and FlexRay for 4-64 bytes frames (**ii**).

Furthermore, with the size of the messages, we include busload in our evaluation by using CANoe, a comprehensive environment for development, testing and analysis of automotive networks. We built CANoe configurations to simulate networks with different number of nodes programmed to generate traffic as imposed by each of the proposed key exchange schemes. The computational time is modeled in the simulation as a delay before the packet is sent. For the pairwise-based approach on CAN-FD we depict the computational time plus the bus delay in Figure 7i. This time, we account for the delays on the bus since the computations are done in parallel and we expect significant reduction in the computational time. Still, the delays on the bus play a less significant role. Clearly, the pairwise approach reduces the time drastically as the number of nodes increases. The pairwise approach can handle 8–16 nodes for the BLS-based protocol version in the same time required by the basic version for 4 nodes. The busload was not significant, always below 1%.

When using FlexRay the communication overhead depends on the length of the communication cycle which is defined as a fixed number of macroticks (MT). The time-triggered nature of the FlexRay protocol assures the predictability of frame arrival times. This is always valid for static frames and also applies to dynamic frames to some extent. We analyze the communication overhead of FlexRay communication during the key exchange process considering a 16 node network, a bit rate of 10M bit/s and a communication cycle with a dynamic segment fixed to 320 MT, a symbol window of 13 MT and a network idle time of 2 MT. We keep the number of slots in the static segment fixed to 20 corresponding to one slot for each of the 16 nodes plus an additional 25% for other traffic of future extensions. We analyze the effect of the static segment size on the time required to complete the key exchange procedure. The static frame payload sizes considered in our analysis along with the communication cycle duration yielded in each case are presented in Table 5. In Figure 7ii we depict the computational time plus transmission time in case of 4, 6, 8, 12, 16, 24, 32, 48 and 64 bytes per frame in the static segment for the pairwise DH-BLSin case of 8 nodes. The computational time dictates the full runtime while transmission time has only a small effect. The runtime is slightly higher than in case of CAN-FD due to the fixed communication cycles as each node must wait for its time-slot, while on CAN-FD messages are sent as soon as they are ready.

## 4. Conclusions

We discussed several key-exchange protocols for in-vehicle networks that rely on four prominent cryptographic constructions: short signatures, identity-based signature, the tripartite Diffie–Hellman key exchange and identity-based key exchanges. Most of these constructions were built on top of bilinear pairings. Computational demands are not low, but the experimental results show that these schemes are ready for practical adoption and, in case of identity-based protocols, they do have the advantage of not requiring public-key certificates which may be troublesome for in-vehicle networks. By moving toward non-pairing-friendly curves, the computational demands lower by five to ten times, while identity-based authentication is still feasible. All the required computations are affordable for high-end cores and future research may come with improvements. Due to obvious performance constraints, it is expected that the security of in-vehicle networks will largely depend on symmetric cryptography and more demanding public-key-based operations will not be performed so often. We expect that key-exchange protocols, such as the ones discussed in our work, are to be performed only during car setup by the manufacturer or in an authorized garage during periodic maintenance services, etc.

## Figures and Tables

**Table 1:** Overview of advantages and disadvantages for the schemes discussed in this work.

Scheme:	DHKE with BLS	DHKE with Paterson IBS	Tripartite-DHKE with Paterson IBS	Wang IBKE	Cao IBKE
Pros:	short signatures	certificate-less	certificate-less	certificate-less	certificate-less
			fewer computations	no signatures	no signatures, no pairings
Cons:	requires certificates	larger signatures	larger signatures	requires additional steps	requires additional steps

**Table 2:** Computational overhead in milliseconds on the Microchip SAM V71 and Infineon TC297.

Microcontroller	BLS			DH Tripartite				Paterson IBS						Wang IBKE					
	GF(p) 160 Bit			GF(2^m^) Curve		GF(p) 512 Bit		GF(2^m^) 384 Bit			GF(p) 512 Bit			GF(2^m^) 384 Bit			GF(p) 512 Bit		
	Gen	Sign	Ver	Share	Key	Share	Key	Gen	Sign	Ver	Gen	Sign	Ver	Gen	Share	Key	Gen	Share	Key
Microchip SAM V71	712.8	106.5	1496	82.04	502	293	978.4	257.6	418.4	1151	4676	1020	2470	159.4	82.04	614.8	3348	293	1152
Infineon TC297	226.3	34.70	451	28.70	142	116	396.4	63.84	147.5	392.4	1690	376.6	904	31.90	28.70	180	1281	116	430.4

**Table 3:** Computational overhead in milliseconds for Cao’s pairing-free IBKE.

Microcontroller	Cao IBKE					
	GF(p) 160-Bit Curve			GF(p) 512-Bit Curve		
	Gen	Share	Key	Gen	Share	Key
Microchip SAM V71	78.2	77.6	379.4	307	290	1168
Infineon TC297	28.3	28.1	135.5	120	114.1	459.1

**Table 4:** Computational overhead and busload (synthetic estimation).

Protocol	Computational Time	Busload
DHKE with BLS (DH-BLS)	$n\left(t_{\mathrm{sDH}}+t_{\mathrm{sBLS}}\right)+\left(n-1\right)\left(t_{\mathrm{eDH}}+t_{\mathrm{vBLS}}\right)$	ℓ(3n+2)
Pairwise DHKE with BLS	$\left(t_{\mathrm{sDH}}+t_{\mathrm{eDH}}+t_{\mathrm{sBLS}}+t_{\mathrm{vBLS}}\right)\log_{2}n$	$2\ell (2^{\log_{2}n+1}-1)$
DHKE with Paterson IBS (DH-Pat)	$n\left(t_{\mathrm{sDH}}+t_{\mathrm{sPat}}\right)+\left(n-1\right)\left(t_{\mathrm{eDH}}+t_{\mathrm{vPat}}\right)$	2ℓ(2n−1)
Pairwise DHKE with Paterson IBS	$\left(t_{\mathrm{sDH}}+t_{\mathrm{eDH}}+t_{\mathrm{sPat}}+t_{\mathrm{vPat}}\right)\log_{2}n$	$3\ell (2^{\log_{2}n+1}-1)$
Tripartite DHKE with Paterson IBS (3DH-Pat)	$n\left(t_{\mathrm{sDH}}+t_{\mathrm{sPat}}+t_{\mathrm{vPat}}\right)+\frac{n}{2}t_{\mathrm{etDH}}$	$7\ell \frac{n}{2}$
Pairwise Tripartite DHKE with Paterson IBS	$\log_{3}n\left(t_{\mathrm{sDH}}+t_{\mathrm{eDH}}+t_{\mathrm{sPat}}+t_{\mathrm{vPat}}\right)$	$3\ell (\frac{3^{\log_{3}n+1}-1}{2})$
Wang IBKE	$nt_{\mathrm{shWang}}+\left(n-1\right)t_{\mathrm{kWang}}$	ℓn
Cao IBKE	$nt_{\mathrm{shCao}}+\left(n-1\right)t_{\mathrm{kCao}}$	2ℓn

**Table 5:** Computation overhead and busload (synthetic estimation).

Payload Size (Bytes)	4	6	8	12	16	24	32	48	64
Cycle duration (µs)	895	935	975	1055	1135	1295	1455	1755	2095
